# Automated volumetric breast density estimation out of digital breast tomosynthesis data: feasibility study of a new software version

**DOI:** 10.1186/s40064-016-2519-4

**Published:** 2016-06-18

**Authors:** Youichi Machida, Ai Saita, Hirofumi Namba, Eisuke Fukuma

**Affiliations:** Kameda Kyobashi Clinic, Tokyo Square Garden 4F, 3-1-1 Kyobashi, Chuo City, Tokyo 104-0031 Japan; Kameda Medical Center, Chiba, Japan; Breast Healthcare Inc., Miyazaki, Japan

**Keywords:** Breast density, Digital breast tomosynthesis, Full-field digital mammography

## Abstract

**Background:**

A new software version of VolparaDensity (Volpara Algorithm version 1.5.1) is capable of calculating volumetric breast density (VBD) using either full-field digital mammography (FFDM) or digital breast tomosynthesis (DBT) images. In this preliminary study, we evaluated the feasibility and consistency of this new automated software.

**Findings:**

Raw data from both DBT and FFDM were acquired from women breast cancer screening at our institution between April and August 2015 using. The DBT and FFDM images obtained under a single compression were collected and VBD was measured using fully automated software. A paired t test was used to analyze differences in the VBD calculated from paired FFDM and DBT images. The correlation coefficient (R value) was calculated and p < 0.05 was considered significant. Dualmodality images were acquired in 160 women; VBD data were available for all but one. There was a significant difference in the VBD of individual breasts calculated from DBT and FFDM and when data were compared per case (<0.001 and p = 0.006, respectively). There were very good to excellent correlations between data from FFDM and from DBT (R = 0.78, p < 0.0001; per breast, R = 0.89, p < 0.0001, per case, R = 0.91, p < 0.0001).

**Conclusions:**

VBD from DBT was well correlated to that from FFDM, though significant differences were observed between the two.

## Background

Digital breast tomosynthesis (DBT) is a limited angle computed tomography technique in which sequential tomographic images through the breast can be reconstructed from projection images obtained at various angles. This technique attempts to increase lesion conspicuity and highlight lesion morphology by minimizing the superimposition of overlying breast tissue that occurs in conventional mammographic images (Qian et al. 2009). Previous investigations have demonstrated that DBT plus full-field digital mammography (FFDM) led to an increase in breast cancer detection rates while decreasing false-positive rates, compared with FFDM alone (Skaane et al. 2013a, b; Ciatto et al. 2013; Rafferty et al. 2013). More recent studies have assessed the potential use of synthetically reconstructed two-dimensional (2D) images, generated from DBT projections, in breast cancer screening. The performance of reconstructed 2D images in addition to DBT was comparable to that of combined FFDM and DBT (Skaane et al. 2014; Gilbert et al. 2015a, b), indicating the possible replacement of FFDM images with synthetic 2D images.

Breast density has been established as an independent risk factor for breast cancer. The high reproducibility and accuracy of volumetric breast density (VBD) evaluation techniques from mammographic images have been reported in several studies (Skippage et al. 2013; Wang et al. 2013; Hammann-Kloss et al. 2014; Machida et al. 2014). Additionally, studies have confirmed a strong correlation between VBD, calculated from mammography, and breast cancer risk (Eng et al. 2014; Brand et al. 2014; Trinh et al. 2015; Schetter et al. 2014).

As DBT is likely play an increasingly important role in breast cancer screening, either as an adjunct to or potential replacement for FFDM, accurate and consistent measures of breast density from both modalities are needed. Results to date comparing the same automated density assessment method on both FFDM and DBT have reported inconsistencies (Tagliafico et al. 2012, 2013).

A new software version of VolparaDensity (Volpara Algorithm version 1.5.1) is capable of calculating VBD using either FFDM or DBT images. Former versions of this software could compute VBD based on FFDM images only. In this preliminary study, we evaluated the feasibility and consistency of this new automated software.

## Methods

VolparaDensity, the software used in this study for the estimation of VBD using FFDM and DBT, was provided by Volpara Health Technologies (Wellington, New Zealand).

### Inclusion criteria of study population

Our institutional review board approved this study and waived the requirement for informed consent. Our study included160 asymptomatic women (age, 22–78 years, mean 44.8; premenopausal, n = 123; postmenopausal, n = 32; menopausal status unknown, n = 5) undergoing breast cancer screening at our institution between April and August 2015. Raw “For Processing” FFDM and DBT images from a dual-modality scanner (MAMMOMAT Inspiration, Siemens Healthcare, Erlangen, Germany) were acquired under a single compression. In the dual-modality protocol, the breast is compressed and then the x-ray tube moves along a limited-angle arc (swing angle, ±25°), which allows for the acquisition of 25 low-dose tomosynthesis projection images (pixel size, 85 µm). These projection images are then used to generate non-overlapping reconstruction images of 1-mm thickness (pixel size, 85 µm). During the same compression, a conventional mammogram (pixel size, 85 µm) is also acquired and output as 2D FFDM images.

### Volumetric breast density

VBD from FFDM was measured from raw images using FDA-cleared fully-automated software (Volpara Algorithm version 1.5.1, Volpara Health Technologies, Wellington, New Zealand). A more detailed description of the algorithm can be found elsewhere (Ng and Lau 2015). Briefly, Volpara uses the pixel signals from the image to determine the X-ray attenuation between the image detector and the X-ray source. From there, it calculates the thickness of adipose versus fibroglandular tissue that must be present between the detector and the X-ray source, by comparing each individual pixel signal to a reference signal of all adipose tissue. The volumes of adipose and fibroglandular tissue are then quantified and summed across the entire breast. VBD is calculated as the percentage of breast volume (BV) that is fibroglandular tissue volume (FTV). Similarly, VBD was measured from DBT raw projections, which are effectively treated by the algorithm as a series of low-dose 2D images. VBD (%) was calculated for each study, each view [i.e. medio-lateral oblique (MLO) and craniocaudal (CC) images] and each breast side. Where the VBD measurement was available for both the MLO and CC views, then the VBD of each breast was taken as the mean of the two views. If only a single view was available, then the VBD of that view was taken as the VBD of that breast. Where the VBD measurement was available for the both breasts, then the VBD of the case was taken as the mean of the two breasts. If only VBD measurement was available for a unilateral breast, then the VBD of that breast was taken as the VBD of that case.

### Statistical analyses

A Paired t-test was used to analyze differences in the VBD calculated from FFDM versus DBT. The correlation coefficient (R value) and its 95 % confidence intervals (CIs) were calculated for variables including the age and calculated VBD. P value <0.05 was considered statistically significant. R values from 0 to 0.25 were regarded as indicating the absence of a correlation. Those from 0.25 to 0.50 were regarded as indicating a poor correlation. Those from 0.50 to 0.75 were regarded as indicating a moderate to good correlation. Finally, R values from 0.75 to 1.0 were regarded as indicating a very good to excellent correlation between the variables (Dawson and Trapp 2004).

## Results

### Study subjects and image data

During the study period, dual-modality images were acquired in 160 women. Both MLO and CC images of both breasts were available in 156 out of the 160 women, only MLO images of both breasts were available in three women, and MLO and CC images of a unilateral breast were acquired (because of prior mastectomy of the contralateral breast) in the remaining one woman. A total of 632 images (319 MLO images and 313 CC images) were processed with Volpara software to obtain VBD measurements from both FFDM and DBT. Of the total images, VBD from FFDM was not successfully calculated in one left MLO view and in 16 CC views (seven left CC and nine right CC) because size of the compressed breast was smaller than lower limit of the application. On the other hand, VBD was successfully calculated in all of the DBT MLO views. The VBD from DBT was unavailable in 13 CC views (five left CC and eight right CC) for the same reason as observed in the case of FFDM. In total, FFDM VBD was available in 616 (316 MLO and 300 CC) out of 632 images (97.5 %), from 316 breasts of 159 women, while DBT VBD was available in 620 (317 MLO and 303 CC) out of 632 images (98.1 %), from 317 breasts of 159 women.

### Comparison of data from FFDM and DBT

A summary of the BV, FTV, and VBD as calculated by Volpara are shown in Table 1. BV calculated from FFDM ranged from 86.1 to 1422.2 cm^3^ (mean 465.3 cm^3^) in MLO images and 90.6–1187.8 cm^3^ (mean 361.1 cm^3^) in CC views, whereas those from DBT ranged from 91.9 to 1425.4 cm^3^ (mean 465.5 cm^3^) in MLO images and 63.8–1185.3 cm^3^ (mean 361.4 cm^3^) in CC views. FTV calculated from FFDM ranged from 11.7 to 187.1 cm^3^ (mean 68.8 cm^3^) in MLO images and 10.9–195.1 cm^3^ (mean 53.8 cm^3^) in CC views, whereas those from DBT ranged from 10.8 to 197.3 cm^3^ (mean 74.8 cm^3^) in MLO images and 7.8–224.5 cm^3^ (mean 53.6 cm^3^) in CC views. There was no significant difference observed in the BV calculated from MLO or CC images obtained from DBT or FFDM (p = 0.10 and 0.41, respectively). The FTV calculated from MLO images was significantly greater on those obtained from DBT compared with FFDM (p < 0.001), whereas no difference was observed for CC images (p = 0.94).

**Table 1:** Range and average of calculated values for breasts from 159 women

		Mean	p
BV (cm^3^)			
MLO images			
FFDM	86.1–1422.2	465.3	0.10
DBT	91.9–1425.4	465.5	
CC images			
FFDM	90.6–1187.8	361.1	0.41
DBT	63.8–1185.3	361.4	
FT (cm^3^)			
MLO images			
FFDM	11.7–187.1	68.8	<0.001
DBT	10.8–197.3	74.8	
CC images			
FFDM	10.9–195.1	53.8	0.94
DBT	7.8–224.5	53.6	
VBD (%)			
MLO images			
FFDM	3.4–37.5	16.7	<0.001
DBT	4.4–37.6	18.0	
CC images			
FFDM	3.3–39.4	17.1	0.94
DBT	4.1–44.7	17.1	
Per breast			
FFDM	3.4–37.6	16.9	<0.001
DBT	4.5–41.1	17.6	
Per case			
FFDM	3.7–34.4	16.9	0.006
DBT	4.7–34.9	17.6	

*BV* breast volume, *CC* cranio-caudal, *DBT* digital breast tomosynthesis, *FFDM* full-field digital mammography, *MLO* medio-lateral oblique

**Fig. 1:**
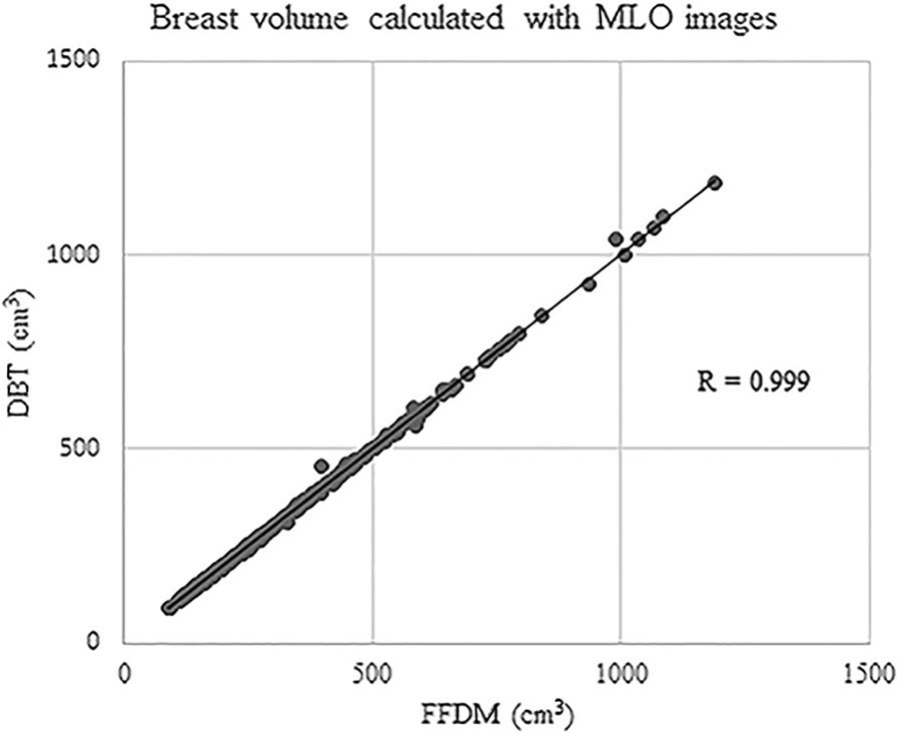
Correlation between breast volume (BV) calculated with medio-lateral oblique (MLO) images of full-field digital mammography (FFDM) and digital breast tomosynthesis (DBT)

**Fig. 2:**
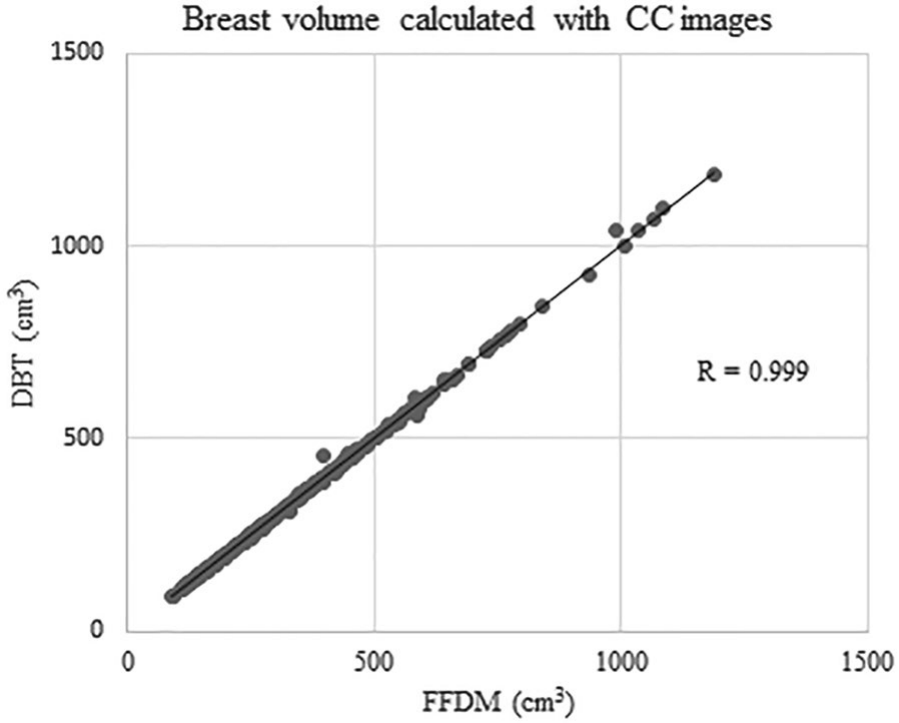
Correlation between BV calculated with craniocaudal (CC) images of FFDM and DBT

**Fig. 3:**
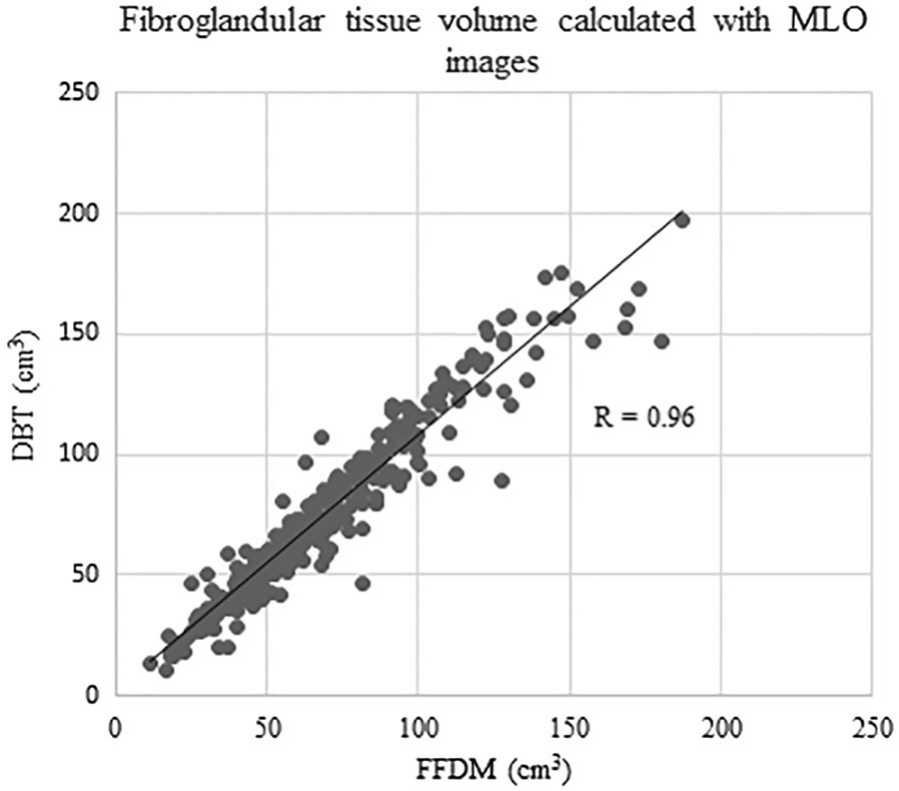
Correlation between fibroglandular tissue volume (FTV) calculated with MLO images of FFDM and DBT

**Fig. 4:**
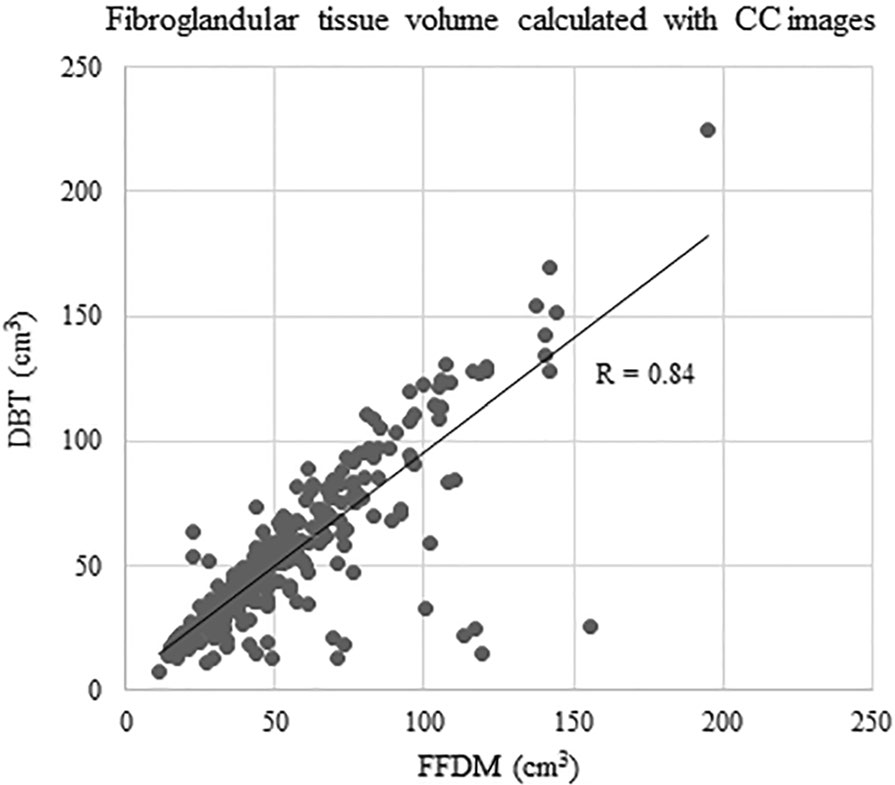
Correlation between FTV calculated with CC images of FFDM and DBT

Very good to excellent correlations were observed between BV and FTV calculated from FFDM and from DBT regardless of image view type (BV from MLO images, R = 0.998, 95 % CI 0.998, 0.999, p < 0.0001; BV from CC images, R = 0.999, 95 % CI = 0.999, 1.000, p < 0.0001; FTV from MLO images, R = 0.96, 95 % CI = 0.95, 0.97, p < 0.0001; FTV from CC images, R = 0.84, 95 % CI 0.80, 0.87, p < 0.0001) (Figs. 1, 2, 3, 4).

**Fig. 5:**
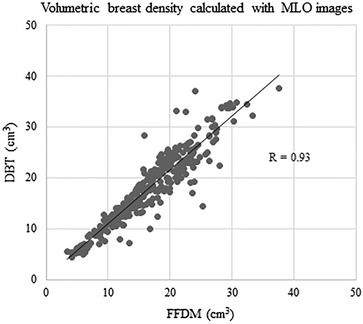
Correlation between volumetric breast density (VBD) calculated with MLO images of FFDM and DBT

**Fig. 6:**
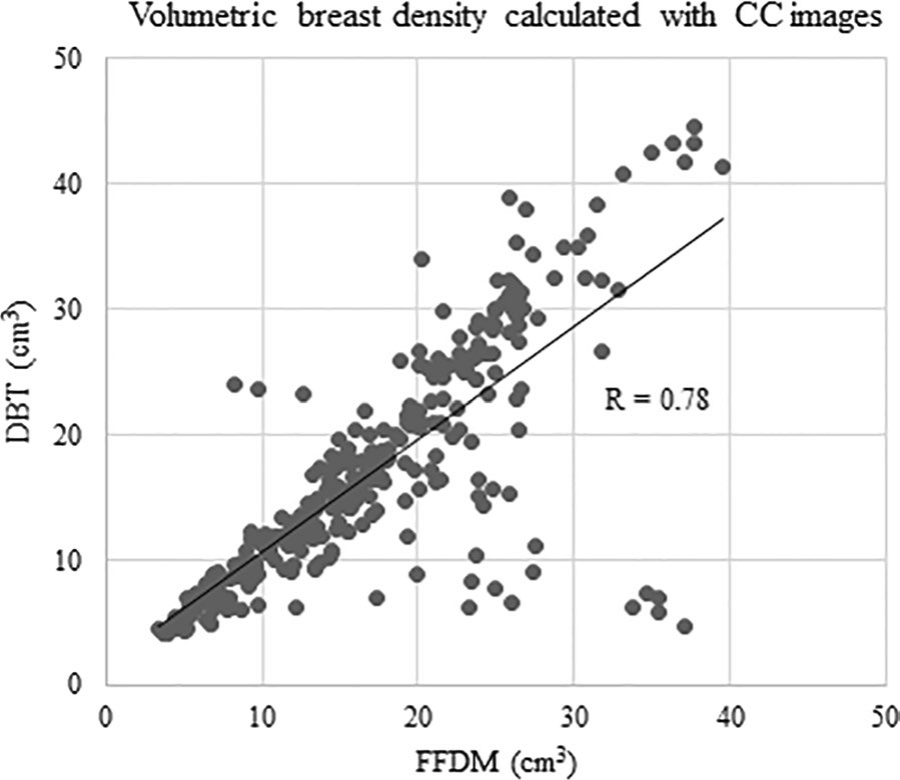
Correlation between VBD calculated with CC images of FFDM and DBT

**Fig. 7:**
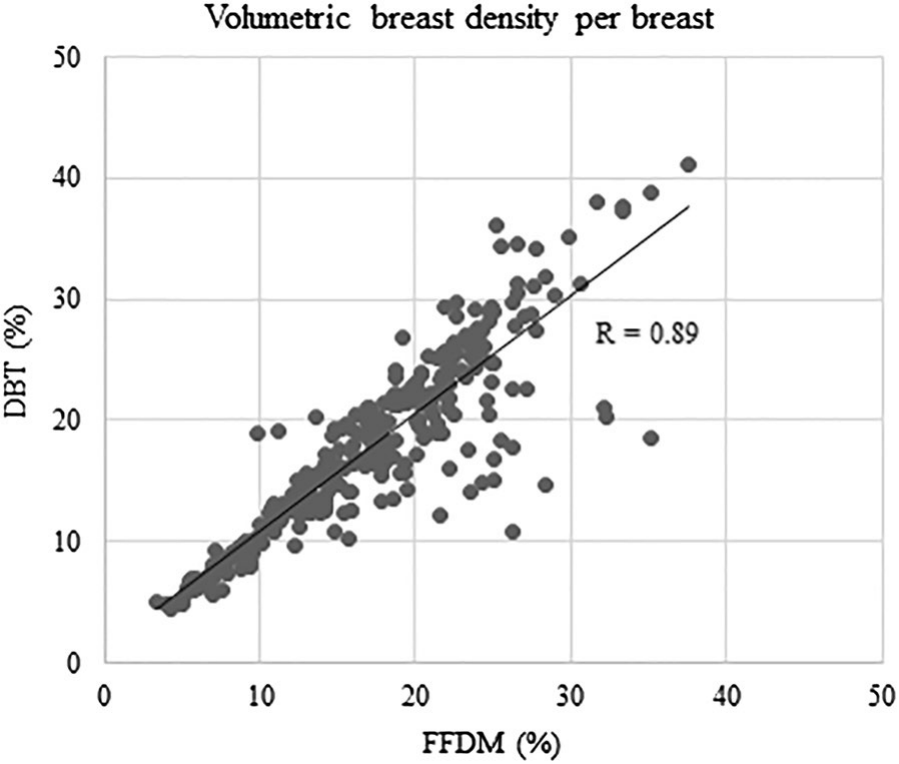
Correlation between VBD per breast from FFDM and DBT

**Fig. 8:**
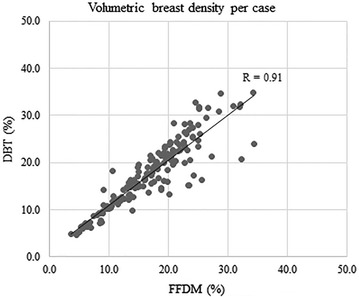
Correlation between VBD per case from FFDM and DBT

VBD calculated from FFDM ranged 3.4–37.5 % (mean 16.7 %) in MLO images and 3.3–39.4 (mean 17.1 %) in CC views, whereas those from DBT ranged 4.4–37.6 % (mean 18.0 %) in MLO images and 4.1–44.7 % (mean 17.1 %) in CC views. VBD calculated from DBT MLO images was significantly greater than that from FFDM MLO images (<0.001), However, there was no significant difference observed in VBD of CC images (p = 0.94). When comparing the VBD calculated from FFDM and DBT either per breast or per case, VBD based on DBT was significantly greater than based on FFDM (per breast, mean 16.9 % and 17.6 % from FFDM and DBT, respectively, <0.001; per case, 16.9 and 17.6 % from FFDM and DBT, respectively, p = 0.006). When the correlations of VBD were assessed by image (MLO and CC images were assessed separately), and by breast, we observed very good to excellent correlations between those calculated from FFDM and DBT (MLO images, R = 0.93, 95 % CI 0.92, 0.95, p < 0.0001; CC images, R = 0.78, 95 % CI 0.73, 0.82, p < 0.0001; per breast, R = 0.89, 95 % CI 0.87, 0.91, p < 0.0001; per case, R = 0.91, 95 % CI 0.87, 0.93, p < 0.0001) (Figs. 5, 6, 7, 8).

### Correlation between age and VBD

**Fig. 9:**
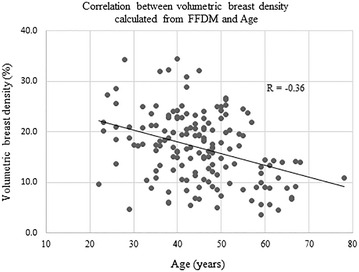
Correlation between age and VBD calculated from FFDM

**Fig. 10:**
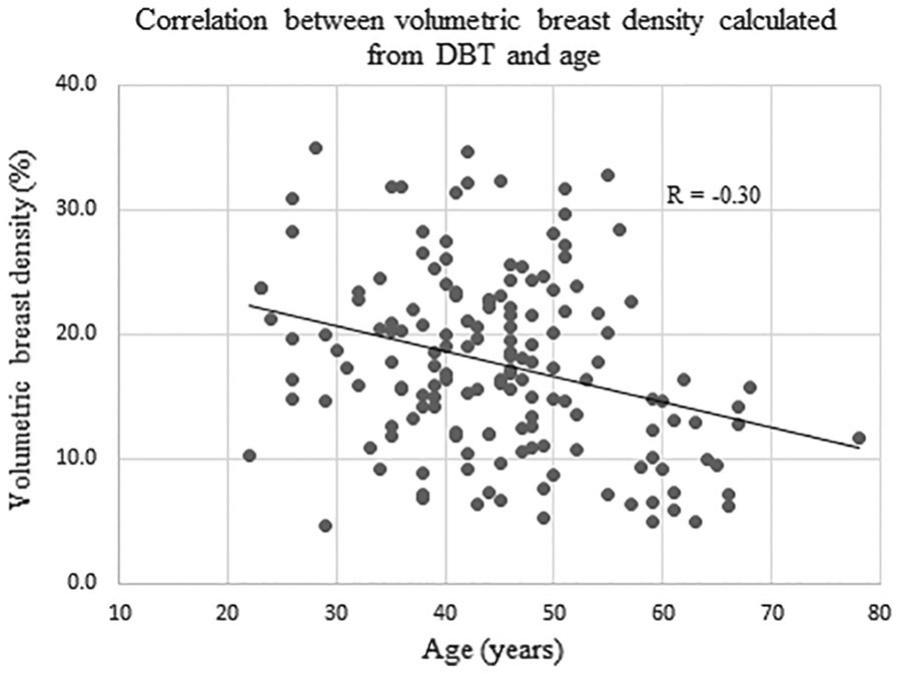
Correlation between age and VBD calculated from DBT

In evaluating the correlation between age and VBD, the average of both breasts was taken as the study VBD, except for the one woman. The VBD of the unilateral breast was used in that patient. There were poor correlations observed between age and VBD, regardless of whether the images were acquired by FFDM or DBT (R = −0.36, 95 % CI −0.49, −0.22, p < 0.0001; R = −0.30, 95 % CI −0.43, −0.15, p = 0.0001, respectively) (Figs. 9, 10).

## Discussion

In accordance with the theoretical advantages of minimizing the superimposition of overlying breast tissue (a key issue for conventional mammography), DBT has been proven to be effective in detecting additional breast cancer occult on conventional mammography and in reducing false-positive cases (Skaane et al. 2013a, b; Ciatto et al. 2013; Rafferty et al. 2013). These benefits are observed across all breast densities, although more so in patients with dense breasts (Gilbert et al. 2015a, b; Ray et al. 2015; Lee et al. 2015; Shin et al. 2015). In addition, the emergence of synthetically reconstructed 2D projection images could make the acquisition of FFDM unnecessary (Skaane et al. 2014; Gilbert et al. 2015a, b). If acquisition of FFDM is to be reduced in the future, accurate breast density information should be obtainable from DBT data, in order to aid both personalized breast cancer screening protocols and breast cancer risk assessment modes that incorporate breast density, and to support epidemiological studies looking at the relationship between breast density and breast cancer outcomes.

Several software applications have been reported to calculate volumetric breast density information from FFDM in a fully automated manner, and with high reproducibility and accuracy (Skippage et al. 2013; Wang et al. 2013; Hammann-Kloss et al. 2014; Machida et al. 2014). In this feasibility study of the commercially available software, Volpara, we first sought to determine whether VBD was calculated successfully from both the DBT and FFDM images. VBD calculation was successfully performed from almost all of the images for both FFDM (97.5 %) and DBT (98.1 %). This high availability of calculated values is necessary for clinical practice.

There was a significant difference observed between the calculated VBD from FFDM versus DBT, with the VBD from FFDM being lower by 1.3 % on average compared with DBT. This difference was consistent with some prior studies which reported that VBD acquired from FFDM was lower than when it was derived from three-dimensional images including DBT or MRI (Wang et al. 2013; van Engeland et al. 2006; Gubern-Merida et al. 2014; Pertuz et al. 2015). In contrast with those reports, Tagliafico et al. (2012, 2013) reported VBD from FFDM was higher than VBD from DBT or MRI. Such significant differences in breast density values calculated using different image or software types should be taken into consideration when the comparing results of future studies that involve breast density values, such as those investigating the relationship between estimated breast density and breast cancer risk.

The availability of VBD estimation using DBT data might be vital in the long term, as increasing use of DBT and reconstructed 2D images may make conventional FFDM image acquisition unnecessary. Therefore, automated breast density should be calculated from DBT images, instead of FFDM alone, and the results checked for self-consistency. The difference in VBD between FFDM and DBT in our population using the new software version was relatively small compared with the differences reported in the studies by Tagliafico et al. (Tagliafico et al. 2013) and by Pertuz et al. (Pertuz et al. 2015) The estimated mean breast density per breast was 16.9 % from FFDM versus 17.6 % from DBT in our current study, a difference of 0.7 %. This favorably compares with 66.1 % from FFDM versus 54.3 % from DBT, a difference of 11.8 % as reported by Tagliafico et al. (2013) and 11.1 % from FFDM versus 19.8 % from DBT, a difference of 8.7 % as reported by Pertuz et al. (2015). The relatively smaller difference between estimated breast density from FFDM and DBT is one advantage of this new software version compared with previously reported methods.

In the assessment of the correlation coefficient between VBD calculated from FFDM and DBT, a very good to excellent correlation was observed in any of MLO by MLO (R = 0.93), CC by CC (R = 0.78), or breast by breast (R = 0.89) comparisons. It should be noted that VBD calculated from MLO images had a higher correlation coefficient than that from CC images, despite the mean VBD being higher in the MLO views compared with the CC views, as mentioned above.

There was a weak correlation observed between the age and VBD acquired both from FFDM and DBT (R = −0.36, −0.30, respectively). The correlation coefficients were similar to a previous study that also evaluated the correlation between the age and volumetric breast density information from a similar population of women in Japan (R = −0.34), calculated using different software (Machida et al. 2014). The similarity of results between the two studies would support the validation of the software used in current study.

Our current study has several limitations. First, this study was a retrospective study with a relatively small-sized data set, and based on FFDM and DBT data acquired by a single scanner and conducted at a single institution. A larger multicenter study with scanners from multiple dual-modality vendors will be necessary for confirming our findings. Second, VBD data acquired from FFDM and DBT were compared with each other without referring to another type of volumetric breast composition method, such as MR imaging. However, the precision of the software used in this study for evaluating FFDM data has been validated in previous studies (Wang et al. 2013; Gubern-Merida et al. 2014), and VBD data calculated from FFDM have already been used as a reference measure in a previous study (Pertuz et al. 2015).

In conclusion, we conducted a feasibility study evaluating a software application for calculation of VBD from DBT and FFDM. VBD acquired from DBT was well correlated to that from FFDM, though statistically significant differences were observed between VBDs computed from the two image types. Such differences should be taken into consideration when comparing the results of future studies investigating breast density for prognostics and disease detection.
